# Genetic Variation in the 3'-Untranslated Region of *NBN* Gene Is Associated with Gastric Cancer Risk in a Chinese Population

**DOI:** 10.1371/journal.pone.0139059

**Published:** 2015-09-24

**Authors:** Ping Sun, Jiangbo Du, Xun Zhu, Chuanli Ren, Lan Xie, Ningbin Dai, Yayun Gu, Caiwang Yan, Juncheng Dai, Hongxia Ma, Yue Jiang, Jiaping Chen, Zhibin Hu, Hongbing Shen, Haorong Wu, Guangfu Jin

**Affiliations:** 1 Department of General Surgery, the Second Affiliated Hospital of Soochow University, Suzhou 215004, China; 2 Department of Pathology, Nanjing Medical University Affiliated Wuxi Second Hospital, Wuxi 214006, China; 3 Department of Epidemiology and Biostatistics, School of Public Health, Nanjing Medical University, Nanjing 211166, China; 4 Jiangsu Key Lab of Cancer Biomarkers, Prevention and Treatment, Collaborative Innovation Center For Cancer Personalized Medicine, Nanjing Medical University, Nanjing 211166, China; 5 Medical Lab, Northern Jiangsu People’s Hospital, Yangzhou 225001, China; 6 Medical Systems Biology Research Center, Tsinghua University School of Medicine, Beijing 100084, China; Medical College of Soochow University, CHINA

## Abstract

*NBN* plays a crucial role in carcinogenesis as a core component for both homologous recombination (HR) and non-homologous end-joining (NHEJ) DNA double-strand breaks (DSBs) repair pathways. Genetic variants in the *NBN* gene have been associated with multiple cancers risk, suggesting pleiotropic effect on cancer. We hypothesized that genetic variants in the *NBN* gene may modify the risk of gastric cancer. To test this hypothesis, we evaluated the association between four potentially functional single nucleotide polymorphisms in *NBN* and gastric cancer risk in a case–control study of 1,140 gastric cancer cases and 1,547 controls in a Chinese population. We found that the A allele of rs10464867 (G>A) was significantly associated with a decreased risk of gastric cancer (odds ratio [OR] = 0.81, 95% confidence interval [95% CI] = 0.71–0.94; *P* = 4.71×10^−3^). Furthermore, the association between A allele of rs10464867 and decreased risk of gastric cancer was more significantly in elder individuals (per-allele OR = 0.72[0.59–0.88], *P* = 1.07×10^−3^), and male individuals (per-allele OR = 0.73[0.62–0.87], *P* = 3.68×10^−4^). We further conducted a haplotype analysis and identified that the *NBN* A_rs10464867_G_rs14448_G_rs1063053_ haplotype conferred stronger protective effect on gastric cancer (OR = 0.76[0.65–0.89], *P* = 6.39×10^−4^). In summary, these findings indicate that genetic variants at *NBN* gene may contribute to gastric cancer susceptibility and may further advance our understanding of *NBN* gene in cancer development.

## Introduction

Gastric cancer is the most common digestive system malignant tumors of the world. China bears the greatest gastric cancer burden of the world. It is estimated that 42.6% new gastric cancer cases and 45.0% deaths of the world were occurred in China in 2012 [1]. *Helicobacter pylori* (HP) infection is the most recognized etiological risk factor for gastric cancer [2]. Dietary and lifestyle habits such as tobacco use, alcohol drinking, intake of salted or smoked food, and low consumption of fresh plant foods were risk factors for gastric cancer development [3]. Although the prevalence of *Helicobacter Pylori* (HP) infection has a high prevalence (40% to 80%) in general population, only a small proportion of infected individuals will develop gastric cancer, suggesting that genetic factors may play an vital role in gastric cancer development [4]. In the past decade, genome-wide association studies (GWAS) have identified several gastric cancer susceptibility loci, including 1q22, 3q13.31, 5p13.1, 8q24.3 and 10q23 [4, 5, 6]. However, GWAS often focus on the peak signals, while the signals with relative moderate significance level may be overlooked. Gene-based analysis strategy therefore can still play an important role in exploring cancer susceptibility, especially for those key genes in carcinogenesis [7].

Genomic instability is an important hallmark of cancer [8]. DNA damaging agents exposure such as chemical exposure, virus infection, etc. may destroy genomic integrity and ultimately result in precancerous lesions or cancer [9]. DNA double-strand breaks (DSBs) is fatal threaten for normal cellular processes and erroneous DNA DSBs repair mechanism regulation may promote cancer development [10]. More interestingly, DSBs were frequently observed in H. pylori-infected human gastric mucosal cells [11]. NBN (NBS1) is a core protein in both DNA DSB repair-homologous recombination (HR) and non-homologous end-joining (NHEJ) pathway in humans. NBN combine with MRE11 and RAD50 can form a MRN protein complex, which is a dynamic macromolecular machine that play a key role in the first step of DNA DSBs repair of both the HR and NHEJ pathway via error recognition or signal transduction [12, 13]. Abnormal expression or complete loss of MRN-complex were frequently observed in multiple cancers [14]. Based on the above evidences researchers considered that genetic variants in NBN may bring to subtle structural alterations of proteins and consequently detrimentally affect cancer susceptibility [15–18]. In the past decade, several *NBN* SNPs were reported to be significantly associated with multiple cancers. Of these significant associations, rs1805794 was first involved in bladder cancer in a Sweden population [19]. Subsequently, a number of studies reported that rs1805794 was also associated with several other cancer including breast cancer [20,21], head and neck cancer [22], prostate cancer [23], hepatic cancer [24], renal cell carcinoma [25], basal cell carcinoma [26]. Furthermore, there were other four SNPs in *NBN* (rs1061302, rs1063054, rs2735383, and rs805794) were reported significantly associated with lung cancer [27, 28, 29], and one (rs376639) was reported to be significantly associated with leukaemia [30]. These evidences collectively suggest a pleiotropic effect of *NBN* SNPs on multiple cancers. However, it is largely unknown for the effect of genetic variants in *NBN* on gastric cancer.

In this study, we hypothesized that the genetic variants in *NBN* may have important implication for gastric cancer susceptibility. Therefore, we conducted a case-control study to investigate the association between four potentially functional single nucleotide polymorphisms (SNPs) in *NBN* gene and gastric cancer risk with 1,140 gastric cancer cases and 1,547 controls in a Chinese population.

## Materials and Methods

### Study subjects

Gastric cancer patients in current study were consecutively recruited from the cities of Yangzhong, Yixing, Yangzhou and Nanjing between January 2004 and December 2011 in Jiangsu Province, Eastern China. Controls were randomly selected from healthy individuals from community-based chronic non-communicable diseases screening program conducted in Changzhou city. After admitting diagnosis, all of the gastric cancer cases were confirmed by histopathologic diagnosis. Furthermore, those patients with any known history of malignancy or having undergone radiotherapy or chemotherapy were excluded. Consequently, a total of 1,140 newly diagnosed gastric cancer cases and 1,547 age and sex frequency-matched controls were included in current study. To obtain the essential personal information such as sex, age, smoking and drinking status, each individual was interviewed by trained investigator with a standard questionnaire. After interview, each subject donated 5 ml peripheral blood, and stored at -20°C. Participants who smoked at least once per day for more than one year were considered as smokers. Who drank more than twice per week for at least one year were defined as drinkers. We summarized the detail information of study subjects in Table 1. Each subject signed an informed consent before interview, and the study was approved by the institutional research ethics committee of Nanjing Medical University.

**Table 1:** Selected characteristics between gastric cancer cases and controls.

Variables	Case (n = 1,140)	Control (n = 1,547)	*P* ^a^
	N (%)	N (%)	
Age	60.99 ± 10.54	60.68 ± 9.21	0.412
Gender			
Male	862 (75.61)	1153 (74.53)	0.522
Female	278 (24.39)	394 (25.47)	
Smoking status			
Never	595 (52.19)	812 (52.49)	0.879
Ever	545 (47.81)	735 (47.51)	
Drinking status			
Never	679 (59.56)	954 (61.67)	0.269
Ever	461 (40.44)	593 (38.33)	
Tumor site			
Cardia	559 (49.04)		
Non-cardia	581 (50.96)		

^a^ T test was used for age and χ^2^ test was used for other binary variables.

### NBN polymorphism selection criteria and genotyping assays

The SNPs were selected based on the NCBI database (http://www.ncbi.nlm.nih.gov/projects/SNP) and the International HapMap Project database (http://hapmap.ncbi.nlm.nih.gov/) according to the following two criteria: 1) located in exon, 3' or 5'untranslated region, and 2) with a minor allele frequency (MAF) of at least 5% in Chinese population. Following these criteria, a total of 10 SNPs were selected. Linkage disequilibrium (LD) analysis with an r^2^ threshold of 0.80 was further applied to filter these SNPs. Finally, 4 SNPs were selected, including rs10464867 (G>A), rs14448 (A>G), rs1063053 (G>A) and rs1063045 (G>A).

Genotyping was performed on the ABI 7900 TaqMan allelic discrimination system (Applied Biosystems, Foster City, CA, USA) without knowing the subjects' grouping situation. The sequence information of primers and probes are shown in S1 Table. Two negative controls were included in each 384-well plate for quality control. The genotypes were determined using the SDS 2.3 Allelic Discrimination Software (Applied Biosystems). The accordance rate of each SNP was 100% for the duplicates of 5% of randomly selected samples.

### Haplotype creation

An analysis based on haplotypes may be advantageous over an analysis based on individual SNPs in the presence of multiple susceptibility alleles, especially when linkage disequilibria between SNPs forming a haplotype are weak [31]. Therefore, we further conducted a haplotype analysis to assess the associations between the haplotype created by these four SNPs and gastric cancer susceptibility. Haplotypes were created by a Confidence Intervals approach, which is the default algorithm in HaploView 4.2 software and is taken from Gabriel et al., 95% confidence bounds on D prime are generated and each comparison is called "strong LD", "inconclusive" or "strong recombination". A block is created if 95% of informative comparisons are "strong LD" [32]. As a result, one block was created and analyzed in current study. Information of Linkage disequilibrium (r^2^ or D’) information among the four SNPs was summarized in S2 Table. Haplotypes were obtained for each sample using the PHASE computer program (ver 2.1), and the haplotype frequencies were estimated via permutation methods [33].

### Statistical analysis

We used chi-square test to compare the distribution differences of categorical variables between cases and controls. A goodness-of-fit chi-square test was used to test the Hardy–Weinberg equilibrium (HWE) between SNPs among the controls. The association analysis between genetic variants and gastric cancer risk, and the evaluation of odds ratios (ORs) and 95% confidence intervals (CIs) were conducted by logistic regression analysis with an adjustment for age, sex, smoking and drinking status. The chi-square-based Q-test was used to test the heterogeneity of effect sizes (ORs and 95% CIs) derived from corresponding subgroups. All statistical analyses were performed with Statistical Analysis System software (version 9.1.3; SAS Institute, Cary, NC).

## Results

Characteristics of 1,140 GC cases and 1,547 controls are summarized in Table 1. No significant differences were detected on age, sex, smoking and drinking status between the cases and controls (*P* = 0.412, 0.522, 0.879 and 0.269, respectively). Genotyping call rates were more than 95% for all four SNPs. The observed genotype frequencies for these SNPs were all in agreement with Hardy–Weinberg equilibrium in the controls (*P* = 0.094 for rs10464867, *P* = 0.095 for rs14448, *P* = 0.145 for rs1063053 and *P* = 0.367 for rs1063045).

The genotype distributions of the four SNPs and its associations with gastric cancer risk are shown in Table 2. Logistic regression analyses revealed that the A allele of rs10464867 were strongly associated with a decreased risk of gastric cancer (per-allele OR = 0.81, 95% CI: 0.71–0.94, *P* = 4.71×10^−3^). In stratification analysis, we further evaluated the associations of the four SNPs on gastric cancer risk in subgroups based on age, gender, smoking, drinking status and tumor sites. A significant difference between subgroups of gender (*P* for heterogeneity = 0.040) were observed for the association of rs10464867 with gastric cancer risk. The association between A allele of rs10464867 and decreased risk of gastric cancer was more significantly in elder individuals (per-allele OR = 0.72[0.59–0.88], *P* = 1.07×10^−3^), and male individuals (per-allele OR = 0.73[0.62–0.87], *P* = 3.68×10^−4^), as well, the G allele of rs14448 shown the same results with rs10464867 (Table 3).

**Table 2:** Association results of four SNPs in *NBN* with gastric cancer risk.

SNP	Case	Control	OR (95%CI) ^a^	*P* value ^a^
	N (%)	N (%)		
rs10464867	n = 1139	n = 1547		
GG	806(70.76)	1029(66.52)	1.00	
GA	306(26.87)	453(29.28)	0.87(0.73–1.03)	0.102
AA	27(2.37)	65(4.20)	0.53(0.33–0.84)	6.35×10^−3^
GA ± AA	333(29.24)	518(33.48)	0.82(0.70–0.97)	0.022
A allele			0.81(0.71–0.94)	4.71×10^−3^
rs14448	n = 1136	n = 1543		
AA	687(60.48)	888(57.55)	1.00	
AG	387(34.07)	549(35.58)	0.91(0.78–1.08)	0.284
GG	62(5.46)	106(6.87)	0.76(0.54–1.05)	0.096
AG ± GG	449(39.52)	655(42.45)	0.96(0.91–1.01)	0.138
G allele			0.89(0.79–1.01)	0.074
rs1063045	n = 1139	n = 1546		
GG	378(33.19)	517(33.44)	1.00	
GA	590(51.80)	739(47.80)	1.10(0.93–1.31)	0.276
AA	171(15.01)	290(18.76)	0.81(0.64–1.02)	0.070
GA ± AA	761(66.81)	1029(66.56)	1.01(0.95–1.06)	0.839
A allele			0.93(0.83–1.04)	0.207
rs1063053	n = 1137	n = 1546		
GG	430(37.82)	584(37.77)	1.00	
GA	552(48.55)	710(45.92)	1.06(0.90–1.25)	0.493
AA	155(13.63)	252(16.30)	0.84(0.66–1.06)	0.134
GA ± AA	707(62.18)	962(62.23)	1.00(0.95–1.05)	0.990
A allele			0.95(0.85–1.06)	0.323

^a^ Derived from logistic regression with an adjustment for age, sex, smoking and drinking status.

**Table 3:** Stratified analysis of associations between rs10464867, rs14448, rs1063053, rs1063045 and gastric cancer risk.

Variables	rs10464867 (G > A)			rs14448 (A > G)			rs1063053 (G > A)			rs1063045 (G > A)		
	OR (95%CI) ^a^	*P* ^a^	*P* ^b^	OR (95%CI) ^a^	*P* ^a^	*P* ^b^	OR (95%CI) ^a^	*P* ^a^	*P* ^b^	OR (95%CI) ^a^	*P* ^a^	*P* ^b^
Age (years)												
< 60	0.91(0.73–1.13)	0.386	0.121	0.93(0.77–1.13)	0.482	0.438	0.90(0.76–1.07)	0.255	0.467	0.88(0.74–1.05)	0.172	0.405
≥ 60	0.72(0.59–0.88)	1.07×10^−3^		0.84(0.71–1.00)	0.049		0.98(0.84–1.14)	0.780		0.97(0.84–1.13)	0.712	
Gender												
Male	0.73(0.62–0.87)	3.68×10^−4^	0.040	0.81(0.70–0.94)	5.89×10^−3^	0.041	0.91(0.80–1.03)	0.142	0.290	0.88(0.77–1.00)	0.048	0.067
Femal	1.05(0.78–1.43)	0.744		1.12(0.85–1.47)	0.420		1.06(0.82–1.36)	0.662		1.15(0.89–1.48)	0.292	
Smoking status												
Never	0.77(0.63–0.94)	0.011	0.458	0.85(0.72–1.01)	0.069	0.547	1.05(0.90–1.23)	0.514	0.036	1.04(0.89–1.22)	0.589	0.023
Ever	0.86(0.70–1.07)	0.172		0.92(0.76–1.12)	0.403		0.82(0.69–0.97)	0.019		0.80(0.68–0.94)	8.22×10^−3^	
Drinking status												
Never	0.80(0.67–0.97)	0.021	0.742	0.89(0.75–1.04)	0.150	1.000	0.96(0.83–1.11)	0.620	0.582	0.92(0.79–1.06)	0.239	1.000
Ever	0.84(0.67–1.05)	0.124		0.89(0.73–1.10)	0.284		0.90(0.75–1.07)	0.223		0.92(0.77–1.10)	0.354	
Tumor site												
Cardia	0.85(0.71–1.02)	0.075	0.447	0.95(0.81–1.11)	0.524	0.243	0.97(0.84–1.12)	0.675	0.529	0.99(0.86–1.14)	0.867	0.198
Non-cardia	0.77(0.65–0.93)	6.10×10^−3^		0.83(0.70–0.97)	0.022		0.91(0.79–1.04)	0.166		0.87(0.76–1.00)	0.052	

^a^ Derived from additive model using logistic regression analysis with an adjustment for age, sex, smoking and drinking status.

^b^ *P* for heterogeneity test based on χ^2^-based Q test.

In haplotype analysis, haplotypes were created for rs10464867, rs14448 and rs1063053 in one block. We then performed association analysis and observed that haplotype GAA and AGG were significantly associated with a decreased risk of gastric cancer risk as compared to haplotype GAG, OR were 0.88(0.77–0.99) and 0.76(0.65–0.89), *P* value were 0.034 and 6.39×10^−4^, respectively (Table 4, S1 Fig).

**Table 4:** Association analysis between haplotypes of and gastric cancer susceptibility.

Haplotype ^a^	Case (N%)	Control (N%)	OR(95% CI) ^b^	*P* ^b^
GAG	39.78	36.10		
GAA	37.81	39.24	0.88(0.77–0.99)	0.034
AGG	15.83	18.84	0.76(0.65–0.89)	6.39×10^−4^
GGG	6.58	5.82	1.02(0.81–1.29)	0.843

^a^ SNP order: rs10464867, rs14448, and rs1063053.

^b^ Derived from logistic regression with an adjustment for age, sex, smoking and drinking status.

## Discussion

The *NBN* gene has been implicated in susceptibility to a high number of cancers, whereas no genetic variants have been reported so far as being associated with gastric cancer. In current study, rs10464867 located in *NBN* was identified to be significantly associated with altered risk of gastric cancer in Chinese population.

DNA double-strand break (DSB) is a particularly important form of DNA damage, which may lead to genetic instability and carcinogenesis [10]. NBN protein plays a pivotal role in the maintenance of genomic stability and was extensively involved in nearly all aspects of the DNA DSB metabolism of NHEJ and HRR DSB repair pathways [11]. Any genetic variants of the *NBN* may have detrimental effects on the DNA damage repair, and consequently may predispose to cancer. In the past decade, a certain number of polymorphisms distributed in *NBN* gene have been reported to be associated with several different kinds of malignancy other than gastric cancer [34]. Our analysis based on TCGA (The Cancer Genome Atlas) data further revealed that the *NBN* mRNA expression level in gastric adenocarcinoma was significantly higher than adjacent normal tissues (S2 Fig), suggesting a potential vital role of *NBN* in gastric carcinogenesis. The current study provided important clues that genetic variants in *NBN* might also be involved in the susceptibility of gastric cancer and further highlight the potential pleiotropic effect of *NBN* SNPs on multiple cancers.

The most robust SNP rs10464867 (G>A) is located in the 3’ UTR of *NBN*, having associated with a decreased risk of gastric cancer. The base change of G to A for rs10464867 may bring to subtle structural alterations of NBN protein and consequently modulation of gastric cancer susceptibility. According to the web-based SNP analysis tool SNPinfo (http://snpinfo.niehs.nih.gov/snpfunc.htm), the base change of G to A for rs10464867 may not only influence an exonic splicing enhancer and result in disequilibrium for different isoforms of NBN, but also may alter binding of mature hsa-miR-216a or hsa-miR-380 to its target mRNA [35]. Functional studies reported that over expression of miR-216a can activates the PI3K/Akt and TGF-β pathways by targeting *PTEN* and *SMAD7*, contributing to hepato carcinogenesis and tumor recurrence in hepatic cell cancer [36]. While miR-380-5p can repress p53 expression via a conserved sequence in the p53 3′ untranslated region (UTR) [37]. Based on the evidences above, we can draw a conclusion that the base change of G to A for rs10464867 may modify the transcriptional regulation of *NBN* and then alter the expression of NBN protein by influence the exonic splicing and miRNA binding. Our further functional annotation analysis based on RegulomeDB revealed that rs2697679 which is in moderate LD (r^2^ = 0.222) with rs10464867 is an eQTL loci of *NBN* [38]. These evidences suggest an important biological implication of rs10464867 for gastric cancer development.

DSB repair capacity represents an important source of variability in genome integrity and thus influence cancer risk [39]. In the past two decades, DSB repair capacity of the normal population have been proved to decline with increased age, and males showed better capacity of DSB repair than females in the same age group [40, 41], suggesting that the inter-gender differences of DSB repair capacity may be partly determined by different genetic mechanisms. In current study, a more pronounced protective effect of rs10464867 was observed in males, which provided a possible explanation for inter-gender differences of DSB repair capacity. The base change of G to A for rs10464867 may alter the expression NBN protein and ultimately influence the NHEJ and HRR DSB repair pathways in males. More interestingly, several association studies also found that SNPs located in DNA repair related genes including *ERCC4*, *ERCC6*, *Ku70m*, *APE1*, *XRCC1*, and *XPD* exhibited a differential effect between genders on gastric cancer susceptibility [42–46].

Some limitations of our study need to be addressed. Firstly, we recruited gastric cancer cases from hospitals and selected controls from communities, which might not well represent the whole population and might result in potential selection bias. Second, we could not access more cases and controls to confirm our findings.

In summary, the present study was designed to search for genetic variants in *NBN* associated with gastric cancer and to test a possible pleiotropic effect of *NBN* SNPs on multiple cancers. Finally, we reported that the *NBN* gene contains genetic variants associated with risk for gastric cancer. Further population based studies combining with functional researches are warranted to illustrate the association and molecular mechanisms of these variants in gastric carcinogenesis.

## Supporting Information

S1 Fig Linkage disequilibrium blocks of tagging SNPs in NBN were created based on the default algorithm is taken from Gabriel et al, Science, 2002, using HaploView software 4.2. The value of r2 of each SNPs pair is shown in the crossing areas.
(DOCX)

S2 Fig The NBN mRNA expression analysis between 29 paired gastric adenocarcinoma and adjacent normal tissues based on TCGA data. (http://cancergenome.nih.gov/).
(DOCX)

S1 Table The primers and probes of genotyping for *NBN* polymorphisms. (DOCX)

S2 Table Linkage disequilibrium (r^2^ or D’) information of the four SNPs. (DOCX)
