# Supplementary material for: Genetic Variation in the 3'-Untranslated Region of NBN Gene Is Associated with Gastric Cancer Risk in a Chinese Population
Source: PLoS One. 2015 Sep 24;10(9):e0139059. doi: 10.1371/journal.pone.0139059 (PMC4581712; doi:10.1371/journal.pone.0139059)
Supplement: S1 Fig — The value of r2 of each SNPs pair is shown in the crossing areas. (DOCX) [file pone.0139059.s001.docx]

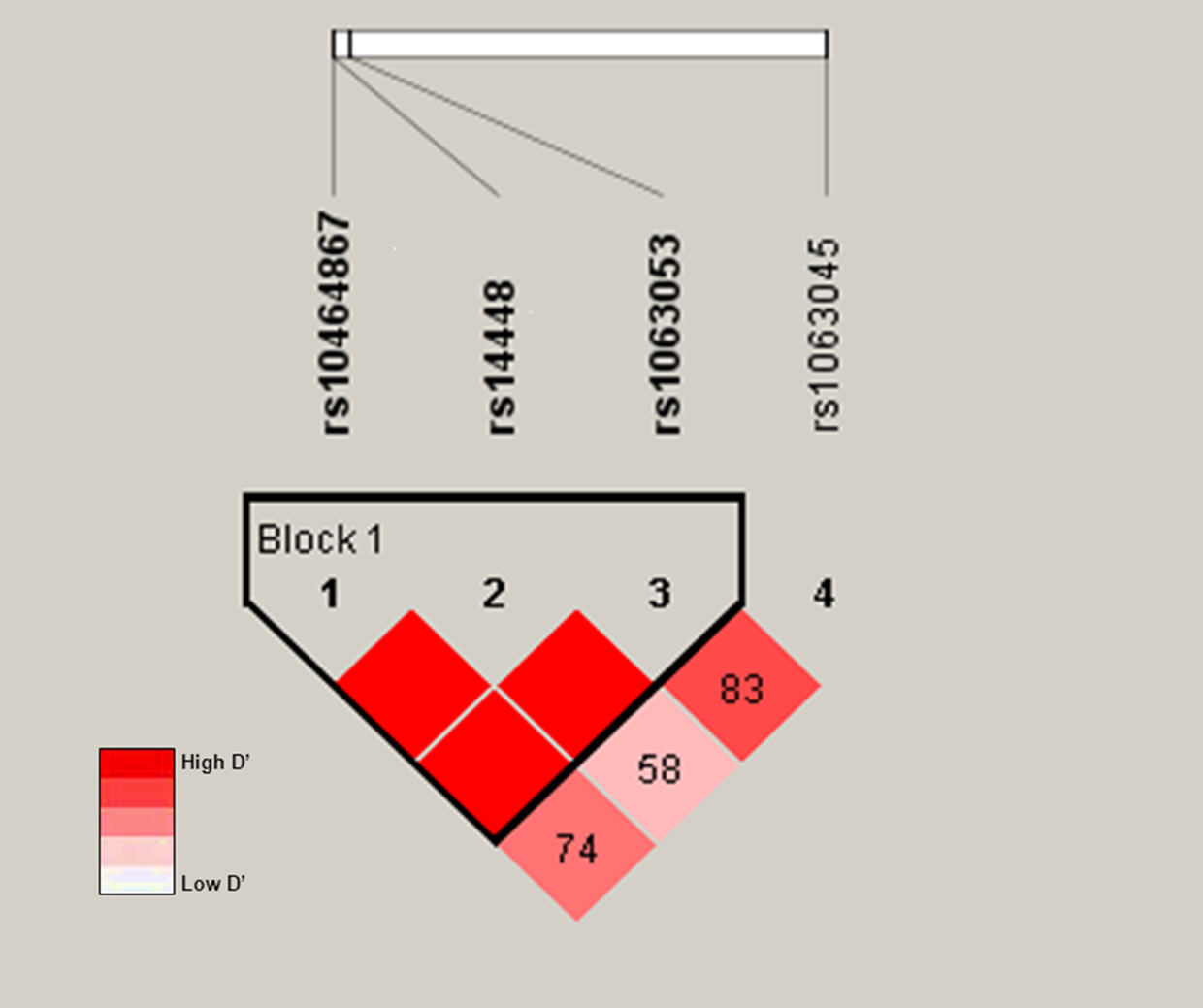


**S1 Fig.** Linkage disequilibrium blocks of tagging SNPs in *NBN* were created based on the default algorithm is taken from Gabriel et al, Science, 2002, using HaploView software 4.2. The value of r^2^ of each SNPs pair is shown in the crossing areas.
