# Supplementary material for: Genetic Variation in the 3'-Untranslated Region of NBN Gene Is Associated with Gastric Cancer Risk in a Chinese Population
Source: PLoS One. 2015 Sep 24;10(9):e0139059. doi: 10.1371/journal.pone.0139059 (PMC4581712; doi:10.1371/journal.pone.0139059)
Supplement: S2 Fig — (http://cancergenome.nih.gov/). (DOCX) [file pone.0139059.s002.docx]

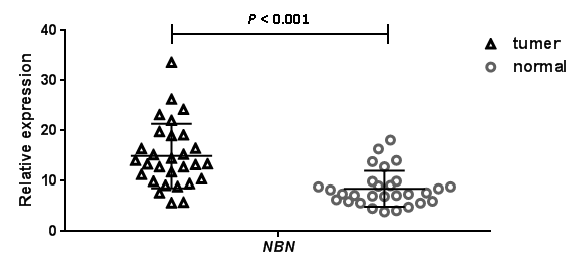


**S2 Fig.** The *NBN* mRNA expression analysis between 29 paired gastric adenocarcinoma and adjacent normal tissues based on TCGA data. (http://cancergenome.nih.gov/)
