# Supplementary material for: Genetic Variation in the 3'-Untranslated Region of NBN Gene Is Associated with Gastric Cancer Risk in a Chinese Population
Source: PLoS One. 2015 Sep 24;10(9):e0139059. doi: 10.1371/journal.pone.0139059 (PMC4581712; doi:10.1371/journal.pone.0139059)
Supplement: S1 Table — (DOCX) [file pone.0139059.s003.docx]

**S1 Table.** The primers and probes of genotyping for *NBN* polymorphisms

| SNPs |  | primers | |  | probes | |
| --- | --- | --- | --- | --- | --- | --- |
| rs10464867 |  | sense | 5’- GACGTAGGTTAAGGTGTATTAAATGCA |  | G allele | FAM-TAATCCCAACTTAAGGAG-MGB |
|  |  | antisense | 5’- GCCTTGAATTACTGTAGCTGCATAG |  | A allele | HEX-TAATCCCAATTTAAGGAGC-MGB |
| rs14448 |  | sense | 5’- CTCCAATTTAACCCCAAGATTTCAGA |  | G allele | FAM-TTGTGAATTACGGATCAG-MGB |
|  |  | antisense | 5’- GGAAAGGATAATTCTTTCAATAAACAGTGT |  | A allele | HEX-TTGTGAATTATGGATCAGG-MGB |
| rs1063045 |  | sense | 5’- TGAGTACGTTGTTGGAAGGAAAAA |  | G allele | FAM-TGTGCCATTCTGATTG-MGB |
|  |  | antisense | 5’- GATTTCGGCTGATCGACTGATC |  | A allele | HEX-TGTGCCATTCTAATTG-MGB |
| rs1063053 |  | sense | 5’- TTCAGGCTTTGTCATTGCATCTTTT |  | A allele | FAM-CAAAATTCTAAATATACGTAAGG-MGB |
|  |  | antisense | 5’- AAGTAGCTAGGACAATGGTGGAAG |  | G allele | HEX-CAAAATTCTAAATGTACGTAAG-MGB |
