# Supplementary material for: Genetic Variation in the 3'-Untranslated Region of NBN Gene Is Associated with Gastric Cancer Risk in a Chinese Population
Source: PLoS One. 2015 Sep 24;10(9):e0139059. doi: 10.1371/journal.pone.0139059 (PMC4581712; doi:10.1371/journal.pone.0139059)
Supplement: S2 Table — (DOCX) [file pone.0139059.s004.docx]

**S2 Table.** Linkage disequilibrium (r^2^ or D’) information of the four SNPs in *NBN*.

| SNPs | rs10464867 | rs14448 | rs1063053 | rs1063045 |
| --- | --- | --- | --- | --- |
| rs10464867 | - | 0.686^a^ | 0.134^a^ | 0.084^a^ |
| rs14448 | 1.000^b^ | - | 0.196^a^ | 0.076^a^ |
| rs1063053 | 1.000^b^ | 1.000^b^ | - | 0.606^a^ |
| rs1063045 | 0.741^b^ | 0.581^b^ | 0.832^b^ | - |

^a^ r^2^; ^b^ D'
